# Supplementary material for: Ethnic diversity fosters the social integration of refugee students
Source: Nat Hum Behav. 2023 Apr 27;7(6):881–91. doi: 10.1038/s41562-023-01577-x (PMC10289893; doi:10.1038/s41562-023-01577-x)
Supplement: Supplementary file 1 — Supplementary Information Text, Figs. 1–7, Tables 1–10 and references. [file 41562_2023_1577_MOESM1_ESM.pdf]

nature human behaviour

Article https://doi.org/10.1038/s41562-023-01577-x

Ethnic diversity fosters the social integration of refugee students

In the format provided by the authors and unedited

Table-of-contents

Appendix A: Complete set of MRQAP results.
Appendix B: Information on the analyzed samples and robustness checks of Fig 1 and Fig 2 results across the samples
Appendix C: Excerpt from student questionnaire
Appendix D: Description of the Multiple Regression Quadratic Assignment Procedure
Appendix E: Robustness check of Fig 3 results
Appendix F: Additional information on the statistical significance of key variables
Supplementary References

Appendix A: Complete set of MRQAP results

Supplementary Table 1 shows how the estimated probabilities of receiving friendship and rejection nominations vary between ethnic majority adolescents (the reference category), first-generation immigrant adolescents, second-generation immigrant adolescents, and refugee adolescents after controlling for the adolescents' gender, age, academic achievement, language skills, and length of stay in Germany. The results stem from two MRQAPs, one having the probability of receiving friendship nominations as the dependent variable and one having the probability of receiving rejection nominations as the dependent variable. The statistical procedure is described in Appendix D. The results demonstrate that refugee students, in comparison to ethnic majority students, have a lower probability of being nominated as friends ($ß$ = -0.067, $p < 0.001$) and a higher probability of being rejected as desk mates ($ß$ = 0.041, $p < 0.001$). Supplementary Table 1 also shows that gender, age, length of stay in Germany, and academic achievement predict the probability of being nominated as a friend, while language skills matter for the probability of being rejected as desk mates by classmates. These results confirm that the findings presented in Fig 1 in the main text are robust to controlling for possible confounders.

Supplementary Table 1. Probabilities of receiving friendship and desk-mate rejection nominations estimated from multivariate MRQAPs.

	Friendship		Rejection	
	Estimate	p	Estimate	p
Intercept (refugee receiver)	0.394	0.000	0.251	0.000
1st-gen immigrant receiver	-0.003	0.053	0.009	0.452
2nd-gen immigrant receiver	0.053	0.000	-0.043	0.000
Native receiver	0.038	0.000	-0.015	0.000
Gender of receiver	-0.002	0.014	-0.027	0.000
Age of receiver	-0.055	0.000	0.062	0.000
Length of stay of receiver	0.008	0.000	-0.002	0.000
Language skills of receiver	0.003	0.137	-0.009	0.000
Academic achievement of receiver	0.013	0.000	-0.019	0.000

$N_{students}$ = 6,390. $N_{classrooms}$ = 304.

Supplementary Table 2 presents the full results from the MRQAPs underlying the results displayed in Fig 3 in the main text.

Supplementary Table 2. Complete results of MRQAP models used to create Fig 3.

	Friendship		Rejection	
	Estimate	p	Estimate	p
Intercept (native → native)	0.442	0.008	0.286	0.265
Diversity	0.012	0.012	-0.087	0.499
Native → refugee	-0.164	<0.001	0.110	<0.001
× diversity	0.086	0.045	-0.058	0.006
Native → 1st-gen immigrant	0.008	0.355	-0.006	0.046
× diversity	-0.085	0.085	0.046	0.304
Native → 2nd-gen immigrant	0.042	0.009	-0.028	0.341
× diversity	-0.092	0.001	0.028	0.359
1st-gen immigrant → native	0.097	0.011	-0.137	0.001
× diversity	-0.144	0.003	0.174	0.004
1st-gen immigrant → refugee	0.077	0.228	-0.223	0.016
× diversity	0.018	0.446	0.318	0.010
1st-gen immigrant → 1st-gen immigrant	0.008	0.245	-0.119	0.142
× diversity	0.044	0.357	0.203	0.115
1st-gen immigrant → 2nd-gen immigrant	0.043	0.070	-0.212	0.004
× diversity	0.021	0.235	0.253	0.006
2nd-gen immigrant → native	0.027	0.068	-0.095	0.001
× diversity	-0.076	0.003	0.126	0.001
2nd-gen immigrant → refugee	-0.061	0.020	0.015	0.230
× diversity	0.010	0.199	-0.003	0.414
2nd-gen immigrant → 2nd-gen immigrant	0.101	0.119	-0.045	0.247
× diversity	-0.131	0.229	0.035	0.224
2nd-gen immigrant → 1st-gen immigrant	-0.111	0.450	0.060	0.203
× diversity	0.127	0.419	-0.066	0.197
Refugee → native	-0.096	<0.001	-0.110	<0.001
× diversity	0.032	0.414	0.076	0.031
Refugee → 1st-gen immigrant	-0.006	0.495	-0.125	0.186
× diversity	0.003	0.411	0.104	0.369
Refugee → 2nd-gen immigrant	0.220	0.007	-0.101	0.045
× diversity	-0.219	0.056	0.094	0.092
Refugee → refugee	0.096	0.001	-0.226	0.004
× diversity	-0.152	<0.001	0.277	0.023
Same country of origin (excl. Germany)	0.191	0.262	0.149	0.187
× diversity	-0.083	0.198	-0.202	0.111
Gender of receiver	0.017	<0.001	-0.032	<0.001
Gender of sender	-0.010	<0.001	0.010	0.001
Same gender	0.248	<0.001	-0.061	<0.001
Age of receiver	-0.003	0.069	0.041	0.001
Age of sender	0.002	0.173	-0.027	<0.001
Age similarity	0.064	<0.001	-0.020	0.015
Length of stay of receiver	-0.001	0.331	0.009	<0.001
Length of stay of sender	0.000	0.419	0.003	0.319
Similarity in length of stay	0.009	<0.001	-0.003	0.458
Language skills of receiver	-0.006	<0.001	-0.002	<0.001
Language skills of sender	0.002	0.215	-0.010	0.001
Similarity in language skills	0.013	<0.001	-0.003	<0.001
Academic achievement of receiver	0.005	0.113	-0.008	0.010
Academic achievement of sender	0.014	<0.001	-0.020	<0.001
Similarity in academic achievement	0.021	<0.001	-0.010	<0.001

N_{students} = 6,390. $N_{\text{classrooms}}$ = 304.

Appendix B: Information on the analyzed samples and robustness checks of Fig 1 and Fig 2 results across the samples

A sample of 39,154 students from 1,807 classrooms was used to create Fig 1. Descriptive statistics of the analyzed variables by immigrant status are provided in Supplementary Table 3. To create Fig 3, we kept only classrooms in the sample that were attended by at least one refugee student. This sample comprised 6,390 students from 304 classrooms, and descriptive statistics of the analyzed variables by immigrant status can be found in Supplementary Table 4. A replication of Fig 1 using this subsample is provided in Supplementary Figure 1.

To create Fig 2, we further restricted our sample to classrooms in which at least 15 students participated in the sociometric part of the questionnaire (i.e., the part in which the students were asked about friendships and desk-mate rejections). This procedure followed the aim of avoiding bias in our results due to nonparticipating peers who would also have nominated refugee students if they had participated. Such selection bias could be severe since, in Fig 2, we report absolute values regarding peer nominations instead of averages. Another reason for our decision was that the average valid class sizes varied across the different diversity levels, with high-diversity settings having the smallest valid class sizes. Given that the number of friendship and desk-mate rejection nominations in the different diversity settings is among the most important messages of Fig 2, we wanted to avoid bias in our results due to the differential participation rates across the different diversity levels. The resulting sample consisted of 5,328 students from 237 classrooms. Descriptive statistics of the analyzed variables by immigrant status are provided in Supplementary Table 5. A replication of Fig 2 using the less restrictive sample that was used to create Fig 3 is provided in Supplementary Figure 2.

In addition, we replicate the findings of Fig 2 by defining “low”, “medium”, and “high” settings based on the whole sample instead of based on the subsample that includes refugee students. This means that the “low” setting now includes those classrooms that are among the third of the classrooms with the lowest levels of diversity in the overall sample, the “high” setting includes classrooms that are among the highest-diversity third of the overall sample, and the ”medium” setting includes the rest of the classrooms. This replication is provided in Supplementary Figure 3 and shows substantively similar tendencies to Fig 2.

We also replicate Fig 2 by using the diversity cut-offs of 0.33 and 0.66 between low- and medium-diversity settings and medium- and high-diversity settings, respectively. These results are shown in Supplementary Figure 4 and demonstrate similar tendencies to the main results.

Finally, we replicate Fig 2 by defining diversity based on the immigrant proportion in the classroom. Here, we defined the three subsamples as the third of all classrooms showing the lowest (up to 26%), the medium (between 26% and 50%), and the highest third of the immigrant proportion in the analysis sample (more than 50%). The replication is provided in Supplementary Figure 5 and shows similar results to those presented in Fig 2.

Supplementary Table 3. Characteristics of analyzed sample.

	Sample (N =39,154)		Ethnic majority (N = 25,659)	Refugee (N = 487)	First-generation immigrant (N = 1,771)	Second-generation immigrant (N = 8,514)	Missing cases
Student characteristics	mean	SD	mean	mean	mean	mean	N
Language skills	0.1	1.8	0.4*	-2.9	-1.4*	-0.3*	0
Mathematics achievement	2.9	1.1	3.0*	2.6	2.8	2.8*	0
Age	15.6	0.6	15.5*	16.6	16.1*	15.5*	361
Length of stay in Germany (in years)	--		--	2.8	8.3*	--	0
	%		%	%	%	%	N
Female	49.0		49.7*	35.9	47.9*	50.5*	0
Classroom characteristics	mean	SD	mean	mean	mean	mean	N
Classroom diversity	0.5	0.2	0.4*	0.7	0.7*	0.7	0
Size	24.9	3.9	24.8*	23.2	24.2	25.3*	0
Network characteristics	mean	SD	mean	mean	mean	mean	N
Density (friendships)	0.4	0.1	0.4*	0.3	0.4	0.4	0
Density (rejections)	0.2	0.1	0.2	0.2	0.2	0.2*	0
Indegrees (friendships)	8.5	3.9	8.8*	5.2	7.7*	8.7*	0
Indegrees (rejections)	4.0	3.0	4.1*	5.4	4.1*	3.8*	0

Student characteristics, classroom characteristics, and network characteristics of the sample used to create Fig 1. Stars indicate significant differences among the given immigrant-status group in comparison to the refugee group ($p < 0.01$). Results from two-tailed analysis of variance (ANOVA) models.

Supplementary Table 4. Characteristics of subsample.

	Sample (N =6,390)		Ethnic majority (N = 3,571)	Refugee (N = 487)	First-generation immigrant (N = 472)	Second-generation immigrant (N = 1,374)	Missing cases
Student characteristics	mean	SD	mean	mean	mean	mean	N
Language skills	-0.9	1.8	-0.3*	-2.9	-2.0*	-1.0*	0
Mathematics achievement	2.7	1.1	2.8*	2.6	2.7	2.7	0
Age	15.8	0.8	15.7*	16.6	16.3*	15.6*	29
Length of stay in Germany (in years)	--		--	2.8	7.1*	--	0
	%		%	%	%	%	N
Female	45.5		46.5*	35.9	46.0*	47.5*	0
Classroom characteristics	mean	SD	mean	mean	mean	mean	N
Classroom diversity	0.6	0.2	0.5*	0.7	0.8*	0.8*	0
Size	24.5	3.7	24.8*	23.2	23.5	24.5*	0
Network characteristics	mean	SD	mean	mean	mean	mean	N
Density (friendships)	0.3	0.1	0.3	0.3	0.3	0.3	0
Density (rejections)	0.2	0.1	0.2	0.2	0.2	0.2	0
Indegrees (friendships)	7.9	3.8	8.4*	5.2	7.0*	8.3*	0
Indegrees (rejections)	4.2	3.0	4.2*	5.4	4.1*	3.7*	0

Student characteristics, classroom characteristics, and network characteristics of the subsample used to create Fig 3. Stars indicate significant differences among the given immigrant-status group in comparison to the refugee group ($p < 0.01$). Results from two-tailed analysis of variance (ANOVA) models.

Supplementary Table 5. Characteristics of subsample.

	Sample (N =5,328)		Ethnic majority (N = 3,119)	Refugee (N = 360)	First-generation immigrant (N = 367)	Second-generation immigrant (N = 1,151)	Missing cases
Student characteristics	mean	SD	mean	mean	mean	mean	N
Language skills	-0.8	1.8	-0.2*	-2.9	-2.0*	-1.0*	0
Mathematics achievement	2.8	1.1	2.8*	2.6	2.7	2.7	0
Age	15.8	0.7	15.7*	16.6	16.3*	15.6*	17
Length of stay in Germany (in years)	--		--	2.8	7.1*	--	0
	%		%	%	%	%	N
Female	45.6		46.6*	36.9	45.0*	46.8*	42
Classroom characteristics	mean	SD	mean	mean	mean	mean	N
Classroom diversity	0.6	0.2	0.5*	0.6	0.7*	0.8*	0
Size	25.1	3.1	25.3	24.6	24.4	25.1	0
Network characteristics	mean	SD	mean	mean	mean	mean	N
Density (friendships)	0.4	0.1	0.4	0.3	0.3	0.4	0
Density (rejections)	0.2	0.1	0.2	0.2	0.2	0.2*	0
Indegrees (friendships)	8.5	3.8	8.9*	5.6	7.6*	8.8*	0
Indegrees (rejections)	4.5	3.1	4.5*	6.1	4.6*	4.0*	0

Student characteristics, classroom characteristics, and network characteristics of the subsample used to create Fig 2. Stars indicate significant differences among the given immigrant-status group in comparison to the refugee group ($p < 0.01$). Results from two-tailed analysis of variance (ANOVA) models.

Supplementary Figure 1. Friendships and desk-mate rejections based on immigration status.

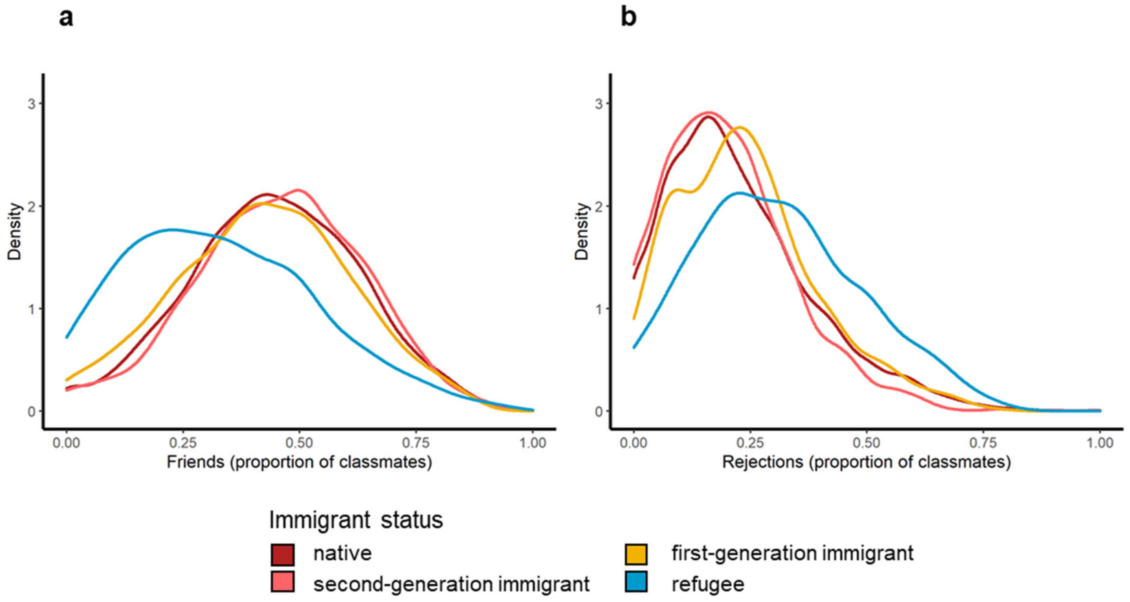

Replication of Fig 1 based on the subsample including only classrooms attended by at least one refugee student. Panel a shows the density plot for friendship and panel b for desk-mate rejection nominations toward refugee adolescents. $N_{students}$ = 6,390. $N_{classrooms}$ = 304.

Supplementary Figure 2. Typical friendship and desk-mate rejection ego networks of refugee students.

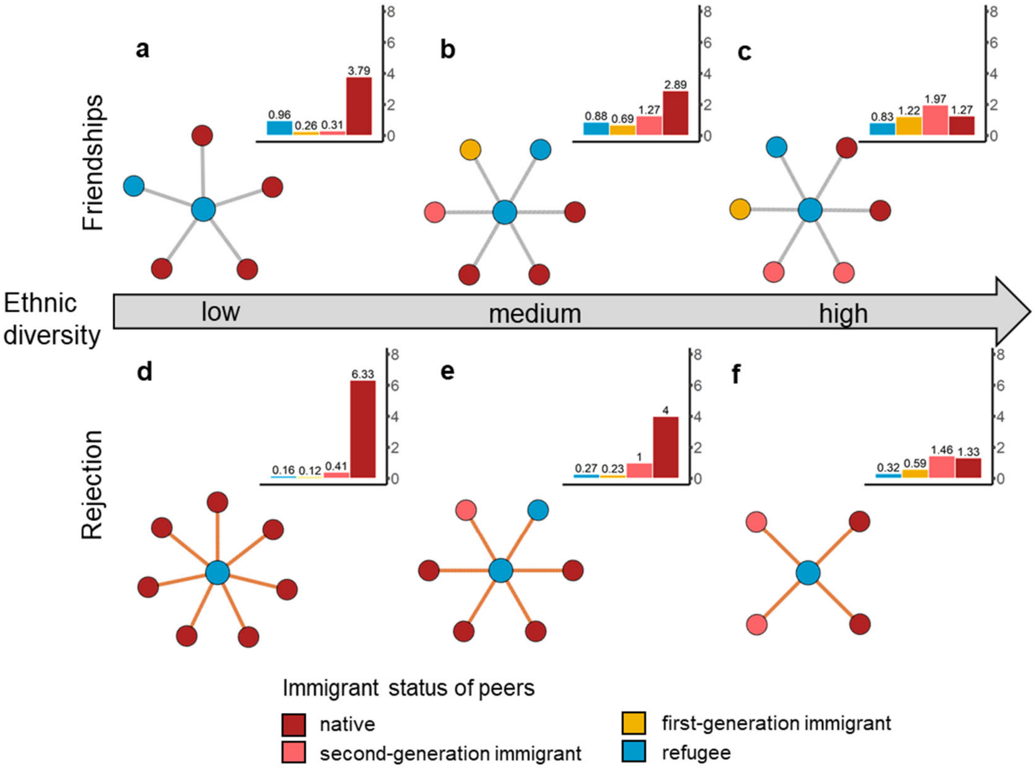

Replication of Fig 2 based on the larger subsample also used to create Fig 3. The bar plots show the rounded average number of friendship (panels a, b, c) and rejection (panels d, e, f) nominations of refugee students by immigrant status group. The network plots map the average number of nominations (e.g., the total number of friends). In addition, the network plots are a proportionate representation of the ethnic composition of nominations of refugee students (e.g., the number of native friends). Note that not every bar can be represented in the network plots by the value of the closest integer to its actual mean due to our primary goal to represent the total number of nominations accurately. This sample includes classrooms in which fewer than 15 students participated in the sociometric questionnaire that included questions about friendships and rejections. N_{students} = 6,390. $N_{\text{classrooms}}$ = 304.

Supplementary Figure 3. Typical friendship and desk-mate rejection ego networks of refugee students.

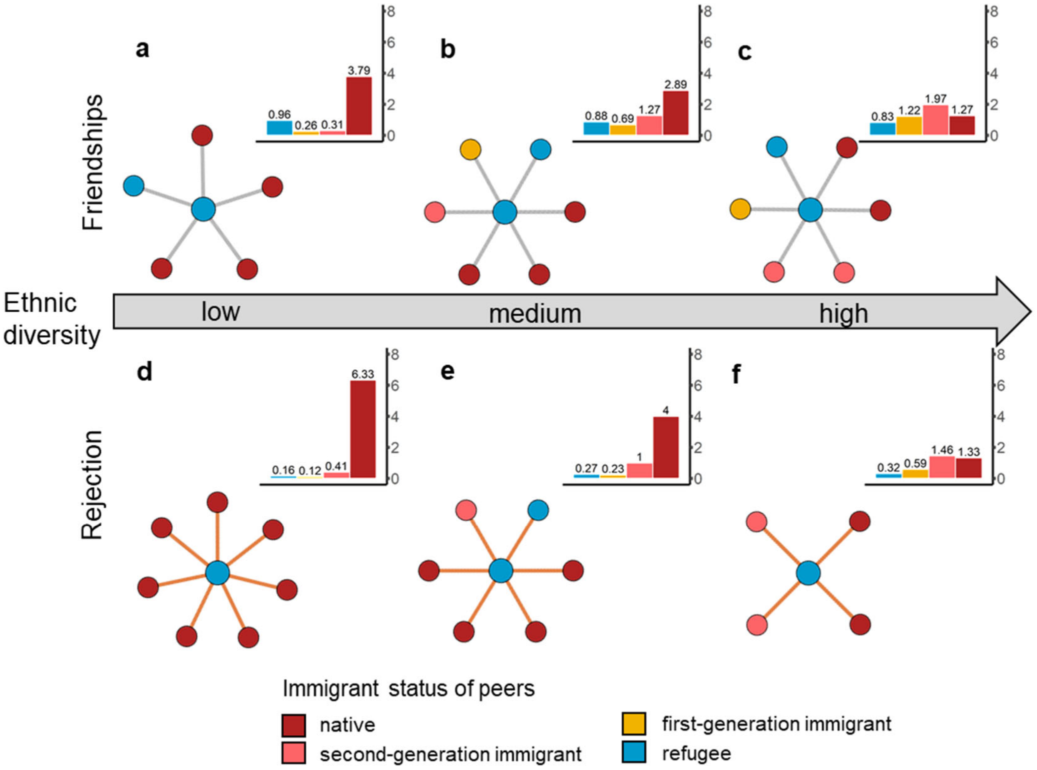

Replication of Fig 2 based on ethnic diversity settings ("low", "medium", "high") calculated from the distribution of diversity in whole sample instead of the subsample that includes refugees. The bar plots show the rounded average number of friendship (panels a, b, c) and rejection (panels d, e, f) nominations of refugee students by immigrant status group. The network plots map the average number of nominations (e.g., the total number of friends). In addition, the network plots are a proportionate representation of the ethnic composition of nominations of refugee students (e.g., the number of native friends). Note that not every bar can be represented in the network plots by the value of the closest integer to its actual mean due to our primary goal to represent the total number of nominations accurately. This sample includes classrooms in which fewer than 15 students participated in the sociometric questionnaire that included questions about friendships and rejections. $N_{students} = 5{,}328$. $N_{classrooms} = 237$.

Supplementary Figure 4. Typical friendship and desk-mate rejection ego networks of refugee students.

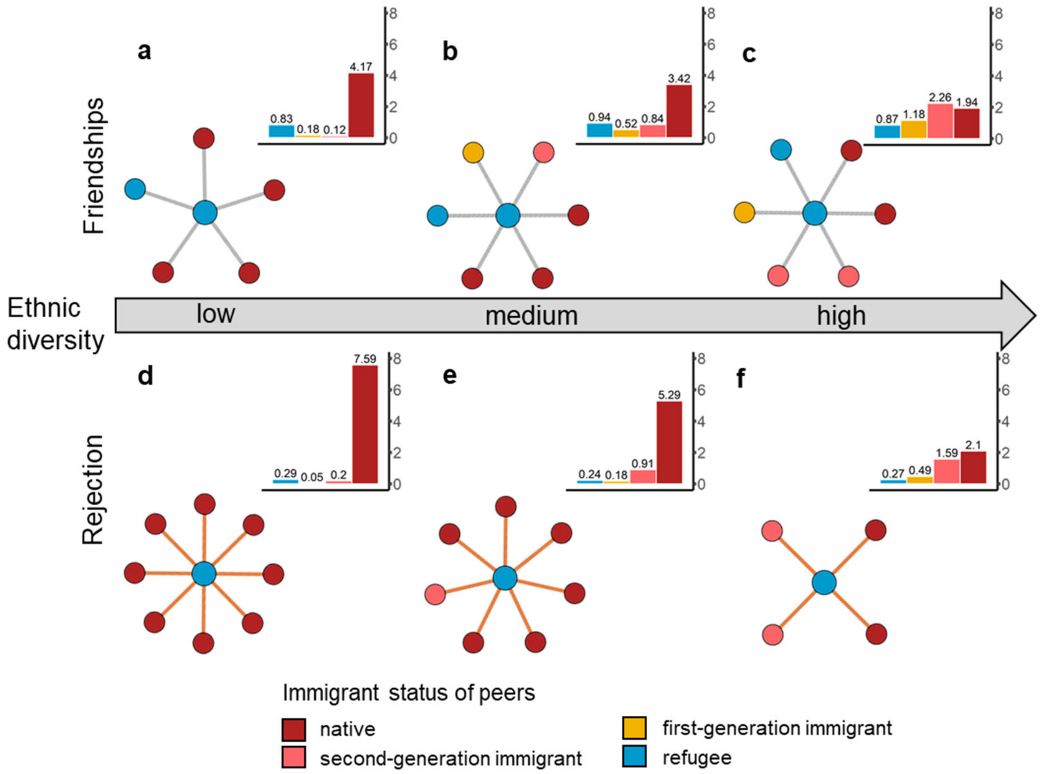

Replication of Fig 2 based using diversity cut-offs 0.33 and 0.66 to create the three diversity categories ("low", "medium", "high"). The bar plots show the rounded average number of friendship (panels a, b, c) and rejection (panels d, e, f) nominations of refugee students by immigrant status group. The network plots map the average number of nominations (e.g., the total number of friends). In addition, the network plots are a proportionate representation of the ethnic composition of nominations of refugee students (e.g., the number of native friends). Note that not every bar can be represented in the network plots by the value of the closest integer to its actual mean due to our primary goal to represent the total number of nominations accurately. This sample includes classrooms in which fewer than 15 students participated in the sociometric questionnaire that included questions about friendships and rejections. $N_{students}$ = 5,328. $N_{classrooms}$ = 237.

Supplementary Figure 5. Typical friendship and desk-mate rejection ego networks of refugee students.

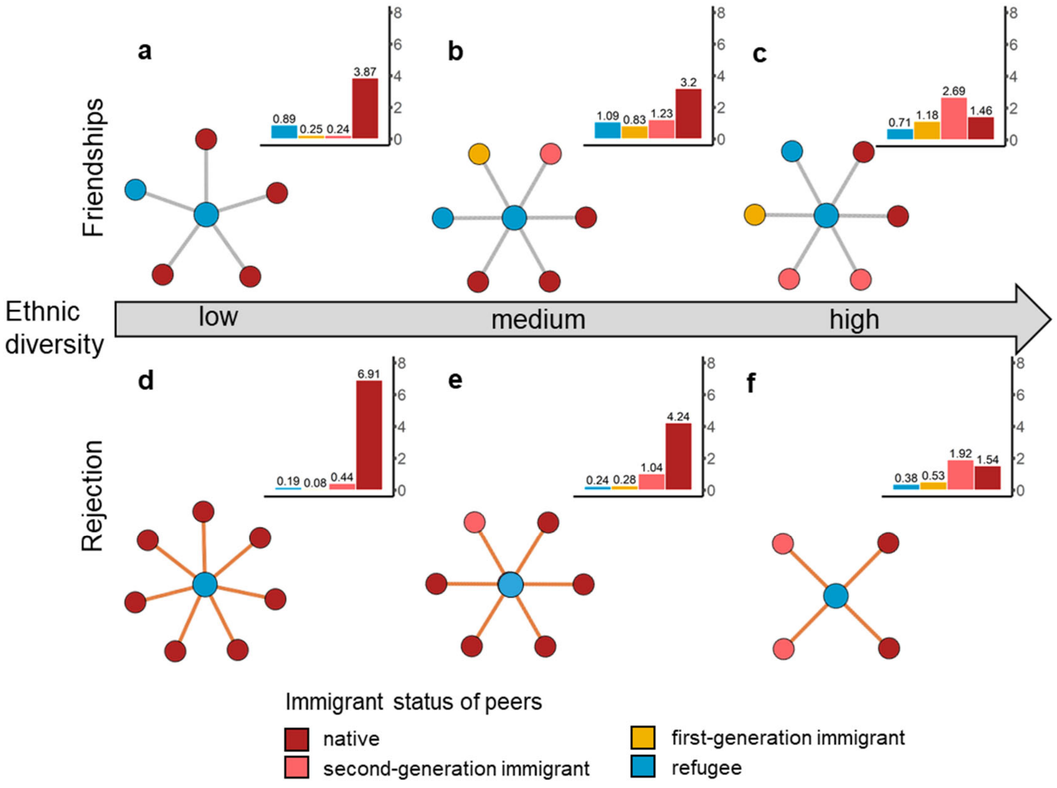

Replication of Fig 2 based using the proportion of immigrants to create the three diversity categories ("low", "medium", "high"). The bar plots show the rounded average number of friendship (panels a, b, c) and rejection (panels d, e, f) nominations of refugee students by immigrant status group. The network plots map the average number of nominations (e.g., the total number of friends). In addition, the network plots are a proportionate representation of the ethnic composition of nominations of refugee students (e.g., the number of native friends). Note that not every bar can be represented in the network plots by the value of the closest integer to its actual mean due to our primary goal to represent the total number of nominations accurately. This sample includes classrooms in which fewer than 15 students participated in the sociometric questionnaire that included questions about friendships and rejections. N_{students} = 5,328. $N_{\text{classrooms}}$ = 237.

Appendix C: Excerpt from student questionnaire.

Supplementary Figure 6. Questionnaire items used to collect sociometric information.

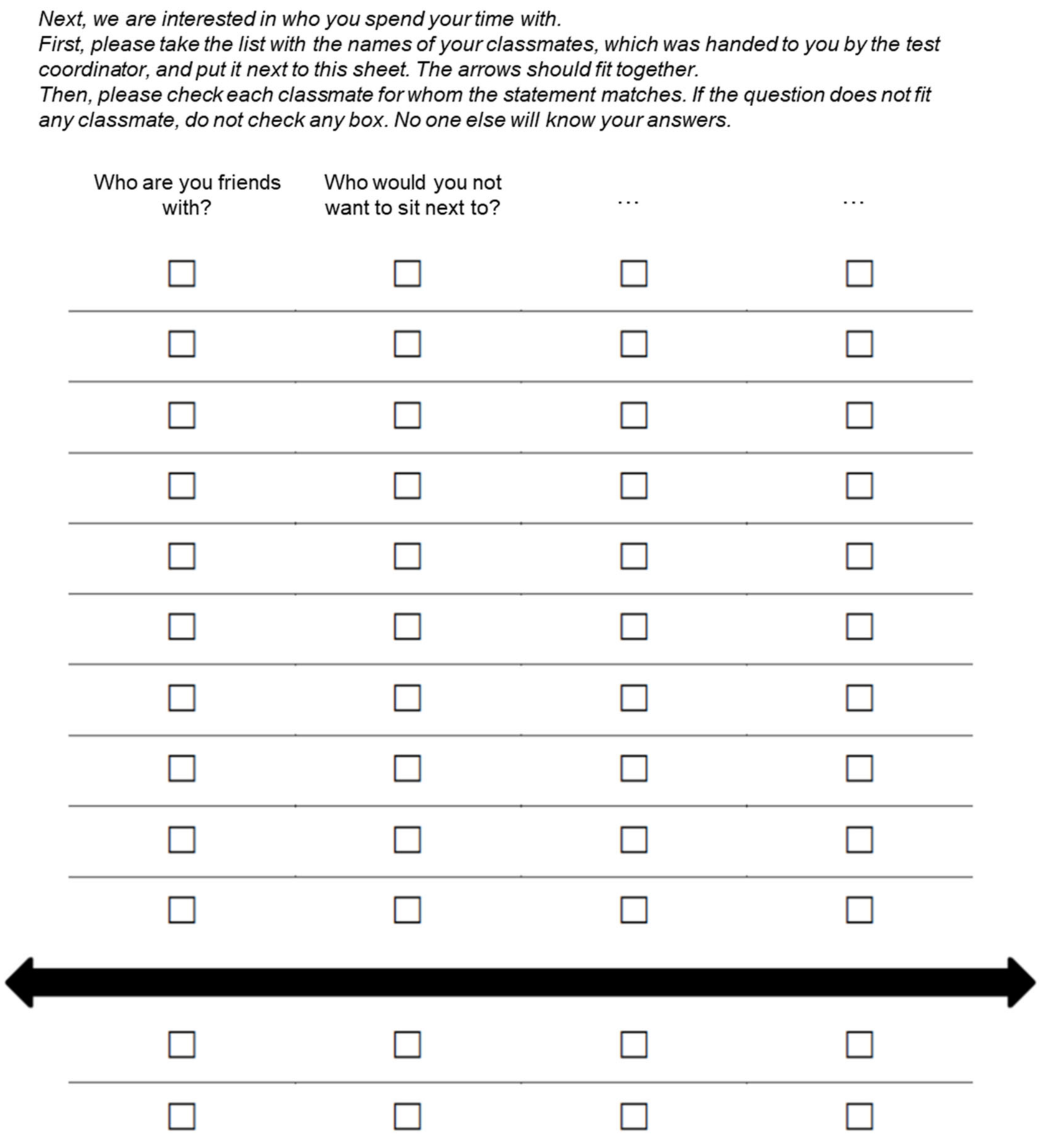

Next, we are interested in who you spend your time with.
First, please take the list with the names of your classmates, which was handed to you by the test coordinator, and put it next to this sheet. The arrows should fit together.
Then, please check each classmate for whom the statement matches. If the question does not fit any classmate, do not check any box. No one else will know your answers.

Who are you friends with?	Who would you not want to sit next to?	…	…
☐	☐	☐	☐
☐	☐	☐	☐
☐	☐	☐	☐
☐	☐	☐	☐
☐	☐	☐	☐
☐	☐	☐	☐
☐	☐	☐	☐
☐	☐	☐	☐
☐	☐	☐	☐
☐	☐	☐	☐
☐	☐	☐	☐
☐	☐	☐	☐

Translated from German.

Appendix D: Description of the Multiple Regression Quadratic Assignment Procedure

Mathematically, the Multiple Regression Quadratic Assignment Procedure (MRQAP) is defined in a similar way to linear regression models, but the data are arranged in matrices instead of vectors:

$$y_{ij} = \beta_0 + \sum_{k=1}^{m} \beta_k (x_{ij}^k) + e_{ij}.$$

Here, y stands for the dependent matrix (that is, friendships or desk-mate rejections), with indexes i and j representing two students from this matrix. Furthermore, m shows the number of independent matrices x^{k} (that is, the number of independent variables). Parameter β^{k} stands for a coefficient, and e_{ij} is an error term. When x^{k} represents a friendship (rejection) network, x_{ij}^k indicates that i names j as a friend (i rejects j). The parameters of MRQAPs can be interpreted as similar to the parameters of a linear regression model because they are estimated with ordinary least squares (OLS) estimators. However, the statistical significance of the estimates is calculated using permutations to take into account dependencies between the observations (1). For this, the OLS regression results are compared to a large number of OLS regression results conducted from “random” data: data for which the dependent matrix y has been permuted. The proportion of “random” regression coefficients that are at least as large as our actual coefficient directly translates to a p-value for the given parameter. We used Y-permuted MRQAPs because they represent the most conservative method to obtain statistical inference among MRQAP models. We conducted 1,000 permutations. Cases with missing information on friendship and/or rejection nominations as well as on any of the predictor variables were excluded from the analyses. To be able to jointly analyze all classrooms in our sample, we performed multigroup QAPs. We used a multigroup MRQAP function an implemented in R by Elmer and Stadtfeld (2).

Appendix E: Robustness check of Fig 3 results

Supplementary Tables 6-10 display several models that serve us to investigate the robustness of our main results. Supplementary Table 6 presents the results from MRQAPs that mimic those presented in Supplementary Table 2 but additionally include classroom fixed effects. Supplementary Table 7 presents the results from MRQAPs that mimic those presented in Supplementary Table 2 but use the proportion of immigrants in a classroom as a measure of diversity instead of the dissimilarity index. Supplementary Table 8 presents the results from MRQAPs that mimic those presented in Supplementary Table 2 but use the proportion of immigrants in a classroom as a measure of diversity instead of the dissimilarity index and additionally consider that diversity differs across the German federal states. Supplementary Table 9 presents the results from MRQAPs in which, in addition to the variables presented in Table 2, we take it into account that students of any immigrant background but with higher socio-economic status (SES; expressed by the parents' highest occupational status) may be more likely to name refugee students as friends and less likely to reject them as desk-mates. Finally, Supplementary Table 10 presents results which extend the Table 2 results by variables accounting for students attending different school tracks to be potentially more or less likely to be friends with or reject refugee students (independent of their own immigrant background).

The diversity-related tendency of nominating refugee students in the robustness models is substantively similar to those in the main model. We see differences from the main results (Supplementary Table 2) in statistical significance regarding two effects: 1) the interaction between native → refugee friendship nominations and diversity in the classroom-dummy model, the immigrant-proportion model, and the classroom-and-federal-state models ($p_{\text{classroom-dummy model}} = 0.084$, $p_{\text{immigrant-proportion model}} = 0.105$, $p_{\text{classroom-and-federal-state model}} = 0.174$, $p_{\text{main model}} = 0.045$; see Supplementary Tables 6, 7, 8, and 2, respectively) and 2) the interaction between refugee → refugee rejections and diversity in the same models ($p_{\text{classroom dummy model}} = 0.095$, $p_{\text{immigrant proportion model}} = 0.100$, $p_{\text{classroom and federal state model}} = 0.115$, $p_{\text{main mode}} = 0.023$). These differences may be due to decreased statistical power (especially in case of the refugee → refugee nominations, since only few classrooms had been attended by more than two refugees). It is important to note, however, that the signs of all of these parameters are the same as the ones in the main models. Therefore, we conclude that the differences in the p-values do not affect our substantive conclusions.

Supplementary Table 6. Results of classroom-fixed-effect MRQAP models.

	Friendship		Rejection	
	Estimate	p	Estimate	p
Intercept (native → native)	0.659	<0.001	0.184	0.327
302 class dummies	yes		yes	
Native → refugee	-0.161	<0.001	0.124	<0.001
× diversity	0.054	0.084	-0.076	0.009
Native → 1st-gen immigrant	0.000	0.488	0.038	0.107
× diversity	-0.078	0.075	-0.021	0.330
Native → 2nd-gen immigrant	0.056	0.002	0.000	0.481
× diversity	-0.108	<0.001	0.000	0.468
1st-gen immigrant → native	0.085	0.012	-0.109	<0.001
× diversity	-0.132	0.012	0.133	0.002
1st-gen immigrant → refugee	0.077	0.226	-0.205	0.007
× diversity	0.000	0.489	0.270	0.007
1st-gen immigrant → 1st-gen immigrant	0.073	0.308	-0.166	0.083
× diversity	-0.043	0.403	0.207	0.065
1st-gen immigrant → 2nd-gen immigrant	0.127	0.080	-0.218	0.004
× diversity	-0.073	0.232	0.250	0.004
2nd-gen immigrant → native	0.044	0.028	-0.072	<0.001
× diversity	-0.095	0.004	0.106	<0.001
2nd-gen immigrant → refugee	-0.114	0.041	0.019	0.363
× diversity	0.064	0.219	0.005	0.459
2nd-gen immigrant → 2nd-gen immigrant	-0.035	0.255	-0.003	0.497
× diversity	0.047	0.237	0.001	0.477
2nd-gen immigrant → 1st-gen immigrant	-0.020	0.407	0.069	0.225
× diversity	0.024	0.408	-0.086	0.204
Refugee → native	-0.083	0.001	-0.085	<0.001
× diversity	-0.014	0.349	0.043	0.082
Refugee → 1st-gen immigrant	0.010	0.469	-0.082	0.180
× diversity	-0.031	0.401	0.024	0.413
Refugee → 2nd-gen immigrant	0.139	0.022	-0.121	0.021
× diversity	-0.100	0.139	0.133	0.044
Refugee → refugee	0.285	0.001	-0.150	0.035
× diversity	-0.429	<0.001	0.145	0.095
Same country of origin (excl. Germany)	0.072	0.162	0.037	0.291
× diversity	0.037	0.349	-0.066	0.203
Gender of receiver	0.011	0.001	-0.034	<0.001
Gender of sender	-0.016	<0.001	0.009	<0.001
Same gender	0.251	<0.001	-0.061	<0.001
Age of receiver	-0.045	<0.001	0.034	<0.001
Age of sender	-0.045	<0.001	-0.035	<0.001
Age similarity	0.064	<0.001	-0.017	0.046
Length of stay of receiver	0.000	0.477	0.007	<0.001
Length of stay of sender	0.000	0.385	0.001	0.223
Similarity in length of stay	0.007	<0.001	-0.001	0.281
Language skills of receiver	-0.006	<0.001	0.003	0.005
Language skills of sender	0.002	0.127	-0.005	<0.001
Similarity in language skills	0.012	<0.001	-0.003	0.002
Academic achievement of receiver	0.004	0.004	-0.005	<0.001
Academic achievement of sender	0.014	<0.001	-0.018	<0.001
Similarity in academic achievement	0.022	<0.001	-0.012	<0.001

$N_{students}$ = 6,390. $N_{classrooms}$ = 304. The diversity main effect is excluded to avoid multicollinearity.

Supplementary Table 7. Results of MRQAP models with immigrant proportion instead of diversity.

	Friendship		Rejection	
	Estimate	p	Estimate	p
Intercept (native → native)	0.441	<0.001	0.256	0.050
Immigrant proportion	0.021	0.044	-0.090	0.170
Native → refugee	-0.144	<0.001	0.099	<0.001
× immigrant proportion	0.083	0.105	-0.060	<0.001
Native → 1st-gen immigrant	-0.015	0.087	0.013	<0.001
× immigrant proportion	-0.076	0.095	0.027	0.080
Native → 2nd-gen immigrant	0.018	0.020	-0.025	0.220
× immigrant proportion	-0.087	<0.001	0.036	0.210
1st-gen immigrant → native	0.061	0.039	-0.106	<0.001
× immigrant proportion	-0.136	0.007	0.191	<0.001
1st-gen immigrant → refugee	0.112	0.036	-0.146	0.010
× immigrant proportion	-0.037	0.188	0.291	0.010
1st-gen immigrant → 1st-gen immigrant	0.043	0.338	-0.129	0.060
× immigrant proportion	-0.018	0.460	0.249	0.030
1st-gen immigrant → 2nd-gen immigrant	0.078	0.094	-0.086	0.260
× immigrant proportion	-0.041	0.351	0.114	0.330
2nd-gen immigrant → native	0.011	0.080	-0.071	<0.001
× immigrant proportion	-0.079	<0.001	0.136	<0.001
2nd-gen immigrant → refugee	-0.084	0.006	-0.009	0.120
× immigrant proportion	0.047	0.386	0.047	0.380
2nd-gen immigrant → 2nd-gen immigrant	-0.008	0.119	-0.059	0.310
× immigrant proportion	-0.001	0.013	0.076	0.220
2nd-gen immigrant → 1st-gen immigrant	0.008	0.449	0.018	0.100
× immigrant proportion	-0.034	0.410	-0.031	0.050
Refugee → native	-0.090	<0.001	-0.095	<0.001
× immigrant proportion	0.035	0.452	0.079	0.090
Refugee → 1st-gen immigrant	0.054	0.344	-0.105	0.010
× immigrant proportion	-0.422	0.128	0.482	0.160
Refugee → 2nd-gen immigrant	0.076	0.044	-0.062	0.250
× immigrant proportion	-0.050	0.221	0.058	0.410
Refugee → refugee	0.080	0.023	-0.061	0.060
× immigrant proportion	-0.165	0.006	0.122	0.100
Same country of origin (excl. Germany)	0.138	<0.001	-0.101	<0.001
× immigrant proportion	-0.015	0.018	0.127	0.020
Gender of receiver	0.017	<0.001	-0.032	<0.001
Gender of sender	-0.010	<0.001	0.011	<0.001
Same gender	0.248	<0.001	-0.061	<0.001
Age of receiver	-0.003	0.047	0.042	<0.001
Age of sender	0.002	0.140	-0.026	<0.001
Age similarity	0.063	<0.001	-0.019	<0.001
Length of stay of receiver	-0.001	0.358	0.009	<0.001
Length of stay of sender	0.000	0.332	0.003	0.270
Similarity in length of stay	0.009	<0.001	-0.003	0.440
Language skills of receiver	-0.006	<0.001	-0.002	<0.001
Language skills of sender	0.002	0.303	-0.010	<0.001
Similarity in language skills	0.012	<0.001	-0.003	<0.001
Academic achievement of receiver	0.005	0.147	-0.008	0.010
Academic achievement of sender	0.014	<0.001	-0.020	<0.001
Similarity in academic achievement	0.021	<0.001	-0.010	<0.001

$N_{students}$ = 6,390. $N_{classrooms}$ = 304.

Supplementary Table 8. Results of MRQAP models with classroom- and state-level immigrant proportions.

	Friendship		Rejection	
	Estimate	p	Estimate	p
Intercept (native → native)	0.442	<0.001	0.271	<0.001
Immigrant proportion of classroom	0.024	0.003	-0.067	0.475
Immigrant proportion of federal state	-0.022	0.003	-0.175	0.004
Native → refugee	-0.145	<0.001	0.115	<0.001
× immigrant proportion of classroom	0.075	0.174	-0.044	0.041
× immigrant proportion of federal state	0.069	0.229	-0.190	0.013
Native → 1st-gen immigrant	0.032	0.493	-0.005	0.006
× immigrant proportion of classroom	-0.030	0.195	0.002	0.095
× immigrant proportion of federal state	-0.504	0.012	0.215	0.429
Native → 2nd-gen immigrant	0.034	0.009	-0.031	0.185
× immigrant proportion of classroom	-0.069	<0.001	0.024	0.229
× immigrant proportion of federal state	-0.158	0.113	0.088	0.423
1st-gen immigrant → native	0.065	0.111	-0.104	0.015
× immigrant proportion of classroom	-0.133	0.002	0.190	0.001
× immigrant proportion of federal state	-0.029	0.145	-0.015	0.043
1st-gen immigrant → refugee	0.152	0.028	-0.182	0.010
× immigrant proportion of classroom	0.009	0.259	0.236	0.014
× immigrant proportion of federal state	-0.454	0.244	0.483	0.211
1st-gen immigrant → 1st-gen immigrant	0.088	0.168	-0.147	0.117
× immigrant proportion of classroom	0.067	0.257	0.212	0.109
× immigrant proportion of federal state	-0.690	0.011	0.274	0.447
1st-gen immigrant → 2nd-gen immigrant	0.123	0.027	-0.088	0.352
× immigrant proportion of classroom	-0.012	0.436	0.101	0.199
× immigrant proportion of federal state	-0.405	0.084	0.062	0.195
2nd-gen immigrant → native	0.022	0.039	-0.069	0.031
× immigrant proportion of classroom	-0.065	<0.001	0.136	<0.001
× immigrant proportion of federal state	-0.111	0.271	0.004	0.082
2nd-gen immigrant → refugee	-0.069	0.018	-0.009	0.074
× immigrant proportion of classroom	0.056	0.412	0.037	0.435
× immigrant proportion of federal state	-0.106	0.464	0.046	0.205
2nd-gen immigrant → 2nd-gen immigrant	-0.013	0.098	-0.104	0.280
× immigrant proportion of classroom	-0.007	0.016	0.040	0.191
× immigrant proportion of federal state	0.050	0.321	0.397	0.117
2nd-gen immigrant → 1st-gen immigrant	0.044	0.351	-0.015	0.122
× immigrant proportion of classroom	-0.012	0.464	-0.067	0.018
× immigrant proportion of federal state	-0.306	0.177	0.350	0.158
Refugee → native	-0.118	<0.001	-0.076	0.003
× immigrant proportion of classroom	-0.007	0.161	0.097	0.019
× immigrant proportion of federal state	0.383	<0.001	-0.200	0.014
Refugee → 1st-gen immigrant	0.065	0.223	-0.159	0.025
× immigrant proportion of classroom	-0.016	0.214	0.070	0.415
× immigrant proportion of federal state	-0.400	0.257	0.545	0.183
Refugee → 2nd-gen immigrant	0.160	0.001	-0.071	0.252
× immigrant proportion of classroom	0.053	0.298	0.041	0.413
× immigrant proportion of federal state	-0.959	0.002	0.156	0.493
Refugee → refugee	0.096	0.022	-0.072	0.110
× immigrant proportion of classroom	-0.161	0.009	0.119	0.115
× immigrant proportion of federal state	-0.083	0.404	0.035	0.357
Same country of origin (excl. Germany)	0.118	<0.001	-0.084	0.033
× immigrant proportion of classroom	-0.029	0.024	0.141	0.007
× immigrant proportion of federal state	0.176	0.449	-0.159	0.174

continued

Supplementary Table 8 continued.

Gender of receiver	0.017	0.001	-0.032	<0.001
Gender of sender	-0.010	<0.001	0.011	<0.001
Same gender	0.248	<0.001	-0.061	<0.001
Age of receiver	-0.009	0.012	0.037	<0.001
Age of sender	-0.004	0.032	-0.031	<0.001
Age similarity	0.063	<0.001	-0.022	0.019
Length of stay of receiver	-0.001	0.397	0.009	<0.001
Length of stay of sender	0.001	0.270	0.003	0.338
Similarity in length of stay	0.009	<0.001	-0.002	0.440
Language skills of receiver	-0.006	<0.001	-0.002	<0.001
Language skills of sender	0.002	0.315	-0.009	<0.001
Similarity in language skills	0.012	<0.001	-0.004	0.004
Academic achievement of receiver	0.005	0.108	-0.008	0.006
Academic achievement of sender	0.014	<0.001	-0.019	<0.001
Similarity in academic achievement	0.021	<0.001	-0.011	<0.001

$N_{students}$ = 6,390. $N_{classrooms}$ = 304.

Supplementary Table 9. Results of MRQAP models controlling for student SES.

	Friendship		Rejection	
	Estimate	p	Estimate	p
Intercept (native → native)	0.443	0.001	0.286	0.241
Diversity	0.012	0.013	-0.087	0.500
Native → refugee	-0.166	0.000	0.113	0.000
× diversity	0.089	0.034	-0.062	0.008
Native → 1st-gen immigrant	0.010	0.365	-0.006	0.050
× diversity	-0.089	0.073	0.046	0.307
Native → 2nd-gen immigrant	0.045	0.012	-0.028	0.385
× diversity	-0.097	0.000	0.028	0.409
1st-gen immigrant → native	0.102	0.016	-0.137	0.001
× diversity	-0.154	0.004	0.174	0.004
1st-gen immigrant → refugee	0.072	0.205	-0.214	0.020
× diversity	0.026	0.459	0.302	0.010
1st-gen immigrant → 1st-gen immigrant	0.020	0.231	-0.118	0.145
× diversity	0.031	0.339	0.202	0.095
1st-gen immigrant → 2nd-gen immigrant	0.047	0.076	-0.212	0.004
× diversity	0.017	0.248	0.252	0.005
2nd-gen immigrant → native	0.030	0.047	-0.095	0.000
× diversity	-0.084	0.002	0.127	0.000
2nd-gen immigrant → refugee	-0.066	0.010	0.021	0.195
× diversity	0.018	0.163	-0.015	0.347
2nd-gen immigrant → 2nd-gen immigrant	0.100	0.118	-0.046	0.275
× diversity	-0.134	0.252	0.036	0.260
2nd-gen immigrant → 1st-gen immigrant	-0.112	0.410	0.060	0.178
× diversity	0.129	0.382	-0.067	0.184
Refugee → native	-0.098	0.000	-0.110	0.000
× diversity	0.035	0.355	0.076	0.038
Refugee → 1st-gen immigrant	0.000	0.492	-0.126	0.127
× diversity	-0.004	0.406	0.104	0.328
Refugee → 2nd-gen immigrant	0.229	0.004	-0.101	0.034
× diversity	-0.232	0.043	0.094	0.085
Refugee → refugee	0.099	0.001	-0.231	0.009
× diversity	-0.150	0.000	0.282	0.022
Same country of origin (excl. Germany)	0.191	0.254	0.151	0.198
× diversity	-0.085	0.211	-0.204	0.139
Gender of receiver	0.017	0.001	-0.032	0.000
Gender of sender	-0.012	0.000	0.010	0.000
Same gender	0.248	0.000	-0.061	0.000
Age of receiver	-0.007	0.048	0.041	0.000
Age of sender	-0.003	0.133	-0.027	0.000
Age similarity	0.064	0.000	-0.020	0.024
Length of stay of receiver	-0.001	0.287	0.009	0.000
Length of stay of sender	0.000	0.433	0.003	0.321
Similarity in length of stay	0.009	0.000	-0.003	0.417
Language skills of receiver	-0.005	0.000	-0.002	0.000
Language skills of sender	0.003	0.115	-0.010	0.000
Similarity in language skills	0.012	0.000	-0.004	0.000
Academic achievement of receiver	0.005	0.090	-0.008	0.009
Academic achievement of sender	0.014	0.000	-0.020	0.000
Similarity in academic achievement	0.021	0.000	-0.010	0.000
SES of sender	-0.001	0.000	0.000	0.002
SES of sender → refugee	0.001	0.017	-0.001	0.010

$N_{students}$ = 6,390. $N_{classrooms}$ = 304.

Supplementary Table 10. Results of MRQAP models controlling for school tracks.

	**Friendship**		**Rejection**	
	Estimate	p	Estimate	p
Intercept (native → native)	0.427	0.424	0.304	0.461
Diversity	0.026	<0.001	-0.092	0.241
Native → refugee	-0.164	<0.001	0.114	<0.001
× diversity	0.082	0.044	-0.060	0.008
Native → 1st-gen immigrant	0.007	0.404	0.012	0.009
× diversity	-0.090	0.033	0.025	0.139
Native → 2nd-gen immigrant	0.043	<0.001	-0.020	0.428
× diversity	-0.101	<0.001	0.024	0.457
1st-gen immigrant → native	0.076	0.019	-0.112	0.002
× diversity	-0.122	0.006	0.154	0.011
1st-gen immigrant → refugee	0.067	0.262	-0.212	0.026
× diversity	0.024	0.500	0.335	0.005
1st-gen immigrant → 1st-gen immigrant	-0.017	0.364	-0.097	0.266
× diversity	0.079	0.483	0.186	0.211
1st-gen immigrant → 2nd-gen immigrant	0.016	0.105	-0.226	0.001
× diversity	0.058	0.381	0.282	0.002
2nd-gen immigrant → native	0.029	0.024	-0.063	0.002
× diversity	-0.083	0.001	0.093	<0.001
2nd-gen immigrant → refugee	-0.079	0.015	0.065	0.039
× diversity	0.021	0.184	-0.050	0.156
2nd-gen immigrant → 2nd-gen immigrant	0.103	0.358	-0.028	0.299
× diversity	-0.138	0.414	0.025	0.215
2nd-gen immigrant → 1st-gen immigrant	-0.126	0.294	0.013	0.206
× diversity	0.154	0.261	-0.011	0.207
Refugee → native	-0.102	<0.001	-0.084	<0.001
× diversity	0.049	0.279	0.042	0.188
Refugee → 1st-gen immigrant	-0.004	0.491	-0.110	0.152
× diversity	0.004	0.394	0.097	0.415
Refugee → 2nd-gen immigrant	0.224	0.002	-0.089	0.034
× diversity	-0.243	0.016	0.097	0.059
Refugee → refugee	0.093	0.001	-0.221	0.012
× diversity	-0.159	<0.001	0.292	0.016
Same country of origin (excl. Germany)	0.166	0.309	0.148	0.232
× diversity	-0.054	0.128	-0.211	0.147
Gender of receiver	0.016	<0.001	-0.033	<0.001
Gender of sender	-0.011	<0.001	0.010	0.001
Same gender	0.248	<0.001	-0.061	<0.001
Age of receiver	-0.011	0.021	0.035	<0.001
Age of sender	-0.003	0.102	-0.035	<0.001
Age similarity	0.065	<0.001	-0.025	0.009
Length of stay of receiver	-0.001	0.327	0.008	<0.001
Length of stay of sender	0.000	0.386	0.003	0.268
Similarity in length of stay	0.009	<0.001	-0.003	0.416
Language skills of receiver	-0.005	<0.001	0.001	<0.001
Language skills of sender	0.003	0.085	-0.007	<0.001
Similarity in language skills	0.013	<0.001	-0.004	<0.001
Academic achievement of receiver	0.005	0.099	-0.008	0.004
Academic achievement of sender	0.014	<0.001	-0.020	<0.001
Similarity in academic achievement	0.021	<0.001	-0.010	<0.001
School track 2 (“Schule mit mehreren Bildungsgaengen”)	0.020	<0.001	-0.016	0.014

continued

Supplementary Table 10 continued.

School track 2 → refugee	-0.010	0.184	-0.064	0.464
School track 3 ("Realschule")	0.001	0.199	-0.019	0.189
School track 3 → refugee	0.000	0.253	-0.051	0.130
School track 4 ("Integrierte Gesamtschule")	-0.006	0.458	0.016	0.420
School track 4 → refugee	0.057	0.080	-0.100	<0.001
School track 5 ("Gymnasium")	0.013	0.320	-0.007	0.085
School track 5 → refugee	0.048	0.048	-0.038	0.018

$N_{students}$ = 6,390. $N_{classrooms}$ = 304. "Hauptschule" is the reference category among the school tracks, preparing students for vocational training and attaining a lower secondary qualification; "Schule mit mehreren Bildungsgaengen" and "Realschule" are intermediate tracks in which student can attain lower and intermediate secondary qualifications; "Integrierte Gesamtschule" is an integrated track in which students can attain all types of qualifications. "Gymnasium" is the academic track, preparing students for a higher secondary qualification and ultimately tertiary education.

Appendix F: Additional information on the statistical significance of key variables

To show visually the significance level of each parameter plotted in Fig 3, we plot these parameter values together with the parameter values in the simulated (“random”) networks (see Supplementary Figure 7). In each subfigure, a dot represents the parameter value in the observed network, and a box plot represents the distribution of the values for the same parameter calculated from the simulated networks. This way, the boxes represent randomness: purely due to features of the network structure, we could expect to see parameter values shown by the box. The error bars represent 95% of the distribution: if the dot does not overlap with its respective box or error bar, it is statistically significant. The further the dot is from the box, the least likely that a parameter estimate of that value would be randomly observed.

Supplementary Figure 7 includes four subfigures. In each of them, we show the parameter values for nominating refugees based on the immigrant status of the sender. In subfigure A, we present the parameters for main effects of nominations of refugees in the friendship models. In subfigure B, we present the parameters for diversity interactions with nominations of refugees in the friendship models. In subfigure C, we present the parameters for main effects of nominations of refugees in the desk-mate-rejection models. In subfigure D, we present the parameters for diversity interactions with nominations of refugees in the desk-mate-rejection models.

Supplementary Figure 7. Statistical significance of parameters capturing nominations of refugees.

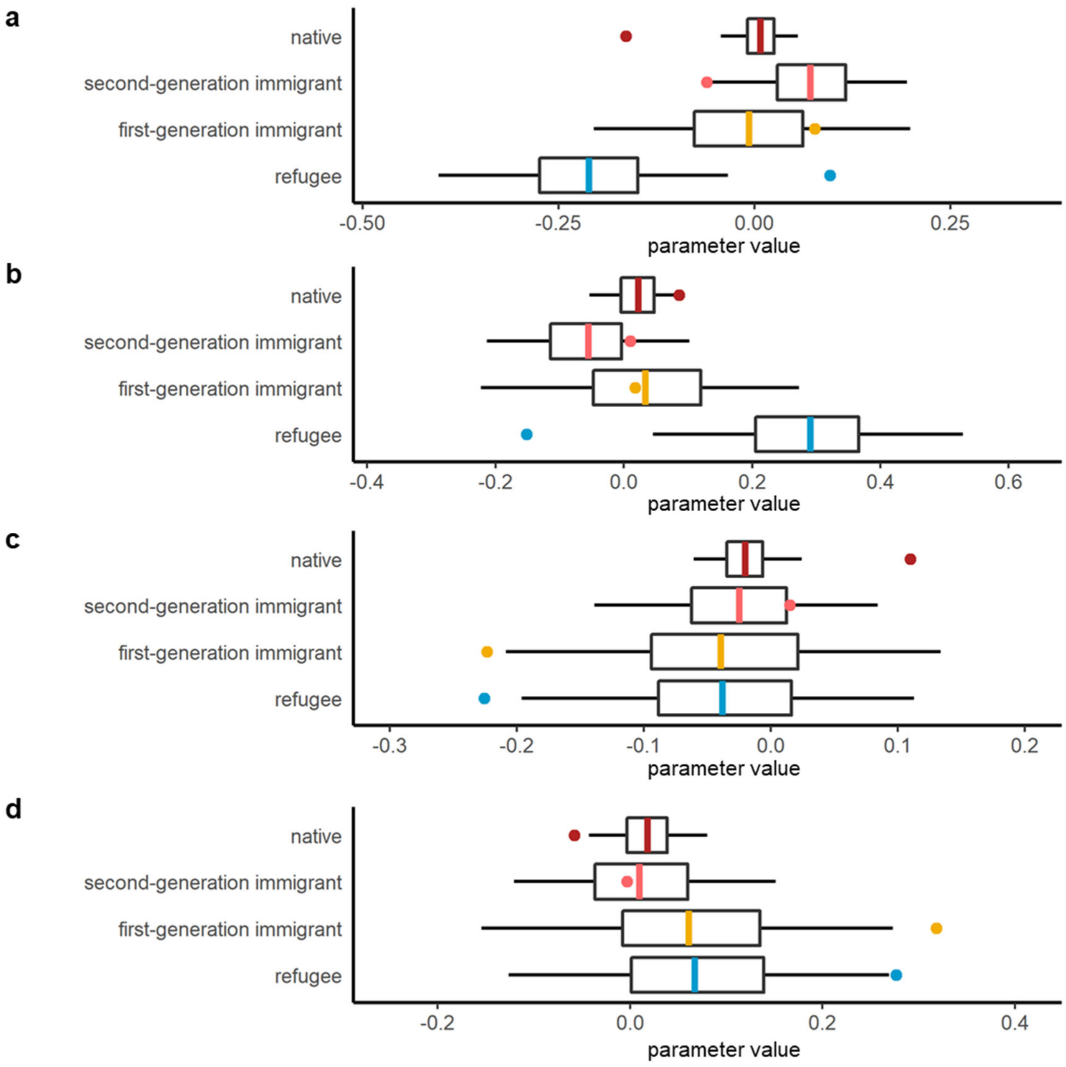

In the subplots of each subfigure, the dots show the parameter estimates for the observed network, whereas the box plots demonstrate features of the distribution of estimates for the simulated networks. Each box represents 50% of the distribution; each error bar represents 95% of the distribution; each vertical line represents the mean of the distribution.
$N_{students}$ = 6,390. $N_{classrooms}$ = 304.
Panel a. Parameters for main effects of nominations of refugees in the friendship models.
Panel b. Parameters for diversity interactions with nominations of refugees in the friendship models.
Panel c. Parameters for main effects of nominations of refugees in the desk-mate-rejection models.
Panel d. Parameters for diversity interactions with nominations of refugees in the desk-mate-rejection models.

Supplementary References

1. D. Dekker, D. Krackhardt, T. A. Snijders, Sensitivity of MRQAP tests to collinearity and autocorrelation conditions. *Psychometrika* **72**, 563–581 (2007).
2. T. Elmer, C. Stadtfeld, Depressive symptoms are associated with social isolation in face-to-face interaction networks. *Scientific reports* **10**, 1–12 (2020).
