# Supplementary material for: Ethnic diversity fosters the social integration of refugee students
Source: Nat Hum Behav. 2023 Apr 27;7(6):881–91. doi: 10.1038/s41562-023-01577-x (PMC10289893; doi:10.1038/s41562-023-01577-x)
Supplement: Supplementary file 2 — Reporting Summary [file 41562_2023_1577_MOESM2_ESM.pdf]

nature portfolio

Corresponding author(s): Zsófia Boda, Georg Lorenz

Last updated by author(s): Feb 3, 2023

Reporting Summary

Nature Portfolio wishes to improve the reproducibility of the work that we publish. This form provides structure for consistency and transparency in reporting. For further information on Nature Portfolio policies, see our Editorial Policies and the Editorial Policy Checklist.

Statistics

For all statistical analyses, confirm that the following items are present in the figure legend, table legend, main text, or Methods section.

n/a	Confirmed	
☐	☒	The exact sample size (*n*) for each experimental group/condition, given as a discrete number and unit of measurement
☐	☒	A statement on whether measurements were taken from distinct samples or whether the same sample was measured repeatedly
☐	☒	The statistical test(s) used AND whether they are one- or two-sided<br>*Only common tests should be described solely by name; describe more complex techniques in the Methods section.*
☐	☒	A description of all covariates tested
☐	☒	A description of any assumptions or corrections, such as tests of normality and adjustment for multiple comparisons
☐	☒	A full description of the statistical parameters including central tendency (e.g. means) or other basic estimates (e.g. regression coefficient) AND variation (e.g. standard deviation) or associated estimates of uncertainty (e.g. confidence intervals)
☐	☒	For null hypothesis testing, the test statistic (e.g. F, t, r) with confidence intervals, effect sizes, degrees of freedom and P value noted<br>*Give P values as exact values whenever suitable.*
☒	☐	For Bayesian analysis, information on the choice of priors and Markov chain Monte Carlo settings
☐	☒	For hierarchical and complex designs, identification of the appropriate level for tests and full reporting of outcomes
☒	☐	Estimates of effect sizes (e.g. Cohen's *d*, Pearson's *r*), indicating how they were calculated

Our web collection on statistics for biologists contains articles on many of the points above.

Software and code

Policy information about availability of computer code

Data collection	No software was used.
Data analysis	The data were analysed using the open source software R (R Core Team, 2019). Custom code supporting this study's findings is available on the Open Science Framework (https://osf.io/as38f/).

For manuscripts utilizing custom algorithms or software that are central to the research but not yet described in published literature, software must be made available to editors and reviewers. We strongly encourage code deposition in a community repository (e.g. GitHub). See the Nature Portfolio guidelines for submitting code & software for further information.

Data

Policy information about availability of data

All manuscripts must include a data availability statement. This statement should provide the following information, where applicable:

- Accession codes, unique identifiers, or web links for publicly available datasets
- A description of any restrictions on data availability
- For clinical datasets or third party data, please ensure that the statement adheres to our policy

The dataset analyzed in the current study was made available for non-commercial research upon application at the Research Data Centre (FDZ) at the Institute for Educational Quality Improvement (IQB) (https://www.iqb.hu-berlin.de/fdz/studies/IQB-BT_2018). For this study, a preliminary internal version was analyzed, which is available from the authors after signing a confidentiality agreement. The two data sets differ in terms of variable names and documentation.

March 2021

Field-specific reporting

Please select the one below that is the best fit for your research. If you are not sure, read the appropriate sections before making your selection.

☐ Life sciences ☒ Behavioural & social sciences ☐ Ecological, evolutionary & environmental sciences

For a reference copy of the document with all sections, see nature.com/documents/nr-reporting-summary-flat.pdf

Behavioural & social sciences study design

All studies must disclose on these points even when the disclosure is negative.

Study description	This is a quantitative study using cross-sectional social network and survey data.
Research sample	The research sample stems from the Trends in Student Achievement Study 2018, conducted by the Institute for Educational Quality Improvement (IQB). The consists of 39,154 students from 1,807 classrooms and is representative of 9th graders at the country level (i.e., for Germany), the federal-state level, and the school-type level. Descriptive statistics of the analyzed variables by immigrant status, including age, sex, and immigrant backgroud, are provided in Supplementary Table 3 in in SI Appendix B. The sample was chosen because it is the largest dataset on refugee students' social networks currently available. Additionally, the data set is representative of 9th graders in Germany.
Sampling strategy	The sample was selected by randomly drawing schools based on the distribution of secondary school types (e.g., academic track, intermediate track, and comprehensive track) in each federal state of Germany. Subsequently, classrooms were randomly drawn in each school (one in academic track secondary schools and two in all other schools). For details on the sampling process, see Stanat et al., 2019).<br><br>Reference:<br>Stanat, P., Schipolowski, S., Mahler, N., Weirich, S. & Henschel, S. IQB Trends in Student Achievement 2018. The Second National Assessment of Mathematics and Science Proficiencies at the End of Ninth Grade. Summary. (Waxmann, 2019a).
Data collection	By the decision of the Standing Conference of the Ministers of Education and Cultural Affairs of the States in the Federal Republic of Germany, participation in the proficiency tests for the IQB Trends in Student Achievement 2018 was compulsory both for students at public schools. The tests were conducted by the International Association for the Evaluation of Educational Achievement (IEA Hamburg). Additionally, paper-pencil questionnaires were handed out by IEA-Hamburg staff and filled out by students, teachers, parents, and school administrators. School administrators provided information about the refugee status of students. The researchers were blind to the study hypotheses during data collection.<br><br>Reference:<br>Stanat, P., Schipolowski, S., Mahler, N., Weirich, S. & Henschel, S. IQB Trends in Student Achievement 2018. The Second National Assessment of Mathematics and Science Proficiencies at the End of Ninth Grade. Summary. (Waxmann, 2019a). Available online at https://www.iqb.huberlin.de/bt/BT2018/Bericht
Timing	The data were collected between April 23 and June 22, 2018 (for details, see Stanat et al. 2019).<br><br>Reference:<br>Stanat, P., Schipolowski, S., Mahler, N., Weirich, S. & Henschel, S. IQB Trends in Student Achievement 2018. The Second National Assessment of Mathematics and Science Proficiencies at the End of Ninth Grade. Summary. (Waxmann, 2019a). Available online at https://www.iqb.huberlin.de/bt/BT2018/Bericht
Data exclusions	The analyzed sample consists of 39,154 students from 1,807 classrooms. For the analyses underlying Fig 3, we used subsamples of classrooms that were attended by at least one refugee student (Fig 3) and where at least 15 students answered the questions about friendships and rejections (Fig 2). These samples comprised 6,390 students from 304 classrooms and 5,328 students from 237 classrooms. Information on these samples and robustness analyses of various samples are provided in SI Appendix B.
Non-participation	Participation in the achievement tests was mandatory in all public schools, resulting in a participation rate of 92.4%. Completing the student questionnaire was voluntary in some federal states and mandatory in others, requiring parental consent in the latter states (82.5% overall participation rate).
Randomization	The participants were not allocated into experimental groups.

Reporting for specific materials, systems and methods

We require information from authors about some types of materials, experimental systems and methods used in many studies. Here, indicate whether each material, system or method listed is relevant to your study. If you are not sure if a list item applies to your research, read the appropriate section before selecting a response.

Materials & experimental systems

n/a	Involved in the study
☒	☐ Antibodies
☒	☐ Eukaryotic cell lines
☒	☐ Palaeontology and archaeology
☒	☐ Animals and other organisms
☐	☒ Human research participants
☒	☐ Clinical data
☒	☐ Dual use research of concern

Methods

n/a	Involved in the study
☒	☐ ChIP-seq
☒	☐ Flow cytometry
☒	☐ MRI-based neuroimaging

Human research participants

Policy information about studies involving human research participants

Population characteristics	Descriptive statistics of the human research participants are provided in SI Appendix B. In the research sample, 49 % of the particpants were female and the average age was 15.6. The classroom social networks contained 24.9 students, on average.
Recruitment	See above. For details on the sampling process, see Stanat, P., Schipolowski, S., Mahler, N., Weirich, S. & Henschel, S. IQB Trends in Student Achievement 2018. The Second National Assessment of Mathematics and Science Proficiencies at the End of Ninth Grade. Summary. (Waxmann, 2019a). Available online at https://www.iqb.huberlin.de/bt/BT2018/Bericht
Ethics oversight	The data collection was part of the educational monitoring strategy ratified by the Standing Conference of the Ministers of Education and Cultural Affairs of the Länder in the Federal Republic of Germany. The German school laws and regulations state that participation in large-scale school assessment studies aiming to assure educational quality (including the IQB Trends in Student Achievement studies, but also the PISA, TIMSS, and PIRLS studies) can be obligatory for schools, school principals, teachers, and students. In accordance with these laws and regulations, the study participants are informed about the general content of the achievement tests and surveys in advance of this monitoring. The Ministries of Education of each federal state approve the data collection, including all of the instruments. This approval procedure considers ethical aspects as well as data protection requirements according to German law. For the 2018 Trends in Student Achievement study (which provides the data used in the present study), the Ministries of Education agreed to waive consent requirements and endorse compulsory participation for the above reasons. However, as school laws differ between the 16 federal states, the exact procedure varied: While participation in the achievement test was mandatory in all states, participation in the questionnaires was mandatory in some states (although students were free to skip questions they did not want to answer) and voluntary in others.

Note that full information on the approval of the study protocol must also be provided in the manuscript.
